# Comparative Analysis of Within-Host Mutation Patterns and Diversity of Hepatitis C Virus Subtypes 1a, 1b, and 3a

**DOI:** 10.3390/v13030511

**Published:** 2021-03-19

**Authors:** Kaho H. Tisthammer, Weiyan Dong, Jeffrey B. Joy, Pleuni S. Pennings

**Affiliations:** 1 Department of Biology, San Francisco State University, San Francisco, CA 94132, USA; pennings@sfsu.edu; 2 BC Centre for Excellence in HIV/AIDS, Vancouver, BC V6Z 1Y6, Canada; edong@cfenet.ubc.ca (W.D.); jjoy@bccfe.ca (J.B.J.); 3 Division of Infectious Diseases, Department of Medicine, University of British Columbia, Vancouver, BC V5Z 3J5, Canada; 4 Bioinformatics Programme, University of British Columbia, Vancouver, BC V6T 1Z4, Canada

**Keywords:** hepatitis C virus, within-host evolution, mutation frequency, nucleotide diversity

## Abstract

Understanding within-host evolution is critical for predicting viral evolutionary outcomes, yet such studies are currently lacking due to difficulty involving human subjects. Hepatitis C virus (HCV) is an RNA virus with high mutation rates. Its complex evolutionary dynamics and extensive genetic diversity are demonstrated in over 67 known subtypes. In this study, we analyzed within-host mutation frequency patterns of three HCV subtypes, using a large number of samples obtained from treatment-naïve participants by next-generation sequencing. We report that overall mutation frequency patterns are similar among subtypes, yet subtype 3a consistently had lower mutation frequencies and nucleotide diversity, while subtype 1a had the highest. We found that about 50% of genomic sites are highly conserved across subtypes, which are likely under strong purifying selection. We also compared within-host and between-host selective pressures, which revealed that Hyper Variable Region 1 within hosts was under positive selection, but was under slightly negative selection between hosts, which indicates that many mutations created within hosts are removed during the transmission bottleneck. Examining the natural prevalence of known resistance-associated variants showed their consistent existence in the treatment-naïve participants. These results provide insights into the differences and similarities among HCV subtypes that may be used to develop and improve HCV therapies.

## 1. Introduction

Hepatitis C virus (HCV) is a 9.6 kb, positive-strand, enveloped RNA virus of the family Flaviviridae [1], causing chronic hepatitis, liver cirrhosis, and hepatocellular carcinoma [2]. Over 71 million people worldwide (1% of the world population) are known to have chronic HCV infection [3]. As most RNA viruses, HCV has a high replication rate and lacks proofreading activity of its RNA-dependent RNA polymerase, which allows it to mutate and exist within the same host (patient) as a “quasispecies”, or swarm of similar variants continuously generating mutations [4]. This high genetic diversity poses significant challenges for antiviral drug and vaccine development [5,6]. Currently, no effective vaccine exists for HCV [7]. A better understanding of viral evolutionary dynamics may improve the treatment and control of HCV.

The diversity level of HCV is extensive, stemming from its high mutation rates, high replication rate, large population size, and short generation time [8]. Its genetic diversity is classified into seven main genotypes and over 67 subtypes, out of which genotype 1 subtype a (1a) accounts for the majority of infections [9]. Different genotypes are characterized by 30–35% divergence at the nucleotide level, and different subtypes within a genotype by less than 15% divergence at the nucleotide level [3,10]. The genome of HCV carries a single open reading frame that encodes three structural and seven non-structural proteins, with a 5′ untranslated region (UTR) at the beginning and a 3′ UTR at the end. The UTRs are known to be extremely conserved within and across genotypes [1,11]. In contrast, the 27 amino acids at the N terminus of the E2 gene display remarkable sequence variation and are characterized as a Hyper Variable Region 1 (HVR1). It is the most variable segment of the HCV genome and is involved in immune escape, as it carries a neutralizing epitope [12].

Characterizing the evolutionary dynamics of viral populations is complex, since viral evolution occurs at different scales, from within hosts (within patients), to between hosts (or between patients), to a global scale, and is likely shaped by different factors. For example, one main difference between within-host and between-host evolutionary dynamics is that between-host evolution stems from repeated bottlenecks from transmission events, but within-host evolution does not [13,14]. Within-host evolution is highly affected by hosts’ heterogeneity, strong selective pressures from hosts’ immune system, and trade-offs such as the virus replication rates and the lifespan of the infected cells [15]. Generally, evolution and diversity at the between-host level are easier to study than within-host evolution, where a large number of accurate individual viral sequences per sample (patient/host) are needed from multiple patients and/or timepoints [16,17]. However, up to 80% of HCV-infected patients develop chronic infection, making HCV one of the most successful persistent human viruses that evades the hosts’ immune system [14]. It is therefore critical to understand within-host evolutionary dynamics and what contributes to the success of HCV, for both scientific and practical purposes, including predicting evolutionary outcomes and developing effective treatment strategies.

Recent advances in next-generation sequencing (NGS) technology have improved our ability to study within-host viral evolution. However, a limited number of studies so far assessed the within-host evolutionary dynamics of HCV, and most studies involved only a small number of patients. For example, Gray et al. [13] estimated the within-host (15 samples) and between-host evolutionary rates across the HCV genome, revealing higher evolutionary rates at the within-host than at the between-host level. Raghwani et al. [18] assessed within-host population dynamics in four patients over time, suggesting within-host HCV evolutionary dynamics are shaped by population structure and strong genetic linkage. We recently analyzed within-host mutations and their fitness costs in 195 patients with HCV 1a [19].

Here, we use NGS data from a large number of treatment-naïve patients to compare within-host evolutionary dynamics of HCV across three different subtypes (1a, 1b, 3a) by assessing patterns of mutation frequencies and genetic diversity. Certain genotypes or subtypes are known to be associated with an elevated treatment failure risk [20,21], and understanding the evolutionary dynamics of different subtypes will help uncover fundamental evolutionary differences between them. We also explore the natural prevalence of known resistance-associated variants (RAVs), taking advantage of our deep-sequencing data. We then examine within-host evolutionary pressures compared to those at the between-host level to gain insights into the differences and similarities between the two.

## 2. Materials and Methods

### 2.1. Viral Samples

Viral RNA samples were obtained and sequenced, as described in Tisthammer et al. [19]. Briefly, plasma samples were obtained from HCV treatment-naïve and viremic HCV/HIV co-infected participants from sites in the Canadian Co-infection Cohort (CCC) [22]. The CCC study included participants with diverse backgrounds, risk profiles, and ethnicities across Canada, including extremely marginalized groups (see Tables 1 and 2 of [22]). As stated [22], the CCC study was approved by all the research ethics boards of the participating institutions and the community advisory committee of the Canadian HIV Trials Network (MUHC BMC-06-006 approved on 17 January 2007). HCV RNA was extracted from 500 µL of plasma. The mean viral load of all samples was 1.68 × 10^7^ (copies/mL), and therefore, the mean RNA templates per sample used for cDNA synthesis was 8.41 × 10^6^ (Supplementary Dataset S1). cDNA was synthesized by an oligo d(A)20 primer, which was further amplified by pangenotypic primers using two nested PCR reactions. The resulting 8991-bp amplicons were sequenced on an Illumina MiSeq platform (Illumina, Inc., San Diego, CA, USA).

All sequence reads of virus isolates were quality-filtered and trimmed using BBTools [23], by following the methods of Tisthammer et al. [19]. We incorporated several processes to increase the confidence in calling minor variants, as well as compared the outcomes of different processes to ensure that results did not change. Briefly, after removing the adapters and contaminants (phix), we applied a strict filtering standard by trimming all bases with a phred score ≦35. Both merged and unmerged reads were mapped to a subtype-specific reference sequence using bwa [24] (AF011753 for subtype 1a, GU133617 for subtype 1b, and AB691595 for subtype 3a), and consensus sequences for each sample were generated using Geneious (v. 11.1.4, Biomatters Ltd., Auckland, New Zealand). The resulting sam files were further filtered using a custom script in R to eliminate potential contaminants and chimeric sequences by removing reads with harmonic d [25]. The same files were converted to bam files using samtools [26] and then to frequency tables of nucleotides at each genomic position using Rsamtools {Morgan:2019wp} in R [27]. After filtering steps and eliminating low-quality samples, the total number of samples for each subtype used in our study were 195 for subtype 1a, 21 for subtype 1b, and 39 for subtype 3a. The frequency tables were further processed to calculate transition and transversion mutation frequencies in two separate ways: (1) minor variants frequencies (MVFs) at each site, and (2) frequencies of mutations relative to each subtype’s consensus-reference sequence (i.e., a consensus sequence of all sample consensus sequences within each subtype, generated by Geneious). Types of mutations, such as whether the mutations result in synonymous/nonsynonymous changes, result in drastic amino acid changes, and create CpG sites, were also identified for each site. For each sample, sites with <1000 read counts were removed.

### 2.2. Data Validation

The sequencing method used for our dataset has high accuracy (99.3%) and precision (99.7%) [28]. We also took additional steps to validate our filtering methods and the resulting datasets, as Illumina Miseq has a sequencing error rate of around 0.1% [29]. We were concerned that if our previous filtering steps did not remove the errors efficiently, mutations observed at low frequencies (<0.001) could actually represent sequencing errors. To evaluate whether this posed a problem, we replaced all mutation frequencies < 0.001 with either NA or zero and assessed the resulting mutation frequency patterns. The results showed consistent patterns. Therefore, we used the original datasets for further analysis.

We also assessed whether (1) viral loads and (2) sequence depths biased observed diversity patterns by comparing nucleotide diversity of patient samples to viral loads and average sequence depths (Supplementary Dataset S1). We found no correlation between diversity and viral loads for all samples, as well as within each subtype (Spearman’s rank correlation test, *p*-value = 0.717–0.935, ρ = −0.060–0.082, Figure S1A). Viral loads did not differ significantly between subtypes for our samples (Wilcoxon rank sum test, *p*-value = 0.380–0.739). We also did not find any significant correlation between average sequence depths and viral loads (Spearman’s rank correlation test, *p*-value = 0.111–0.914, ρ = −0.350–0035, Figure S1B). The average sequence depth of all samples was 6115.0 (±341) per site, with no significant differences in average read depths between subtypes (subtypes 1a = 6178 ± 427, 1b = 5891 ± 510, 3a = 5919 ± 590, Wilcoxon rank sum test, *p*-value = 0.595–0.817). Therefore, all samples were included in the analysis.

### 2.3. Data Analysis

**Figure 1:**
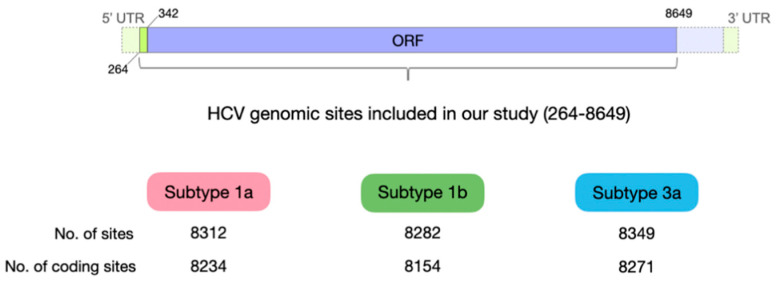
Schematic showing the hepatitis C virus (HCV) genomic sites used in the study. Purple color in the diagram on the top represents the coding region of the HCV genome, which consists of one large open reading frame (ORF) for polyproteins, and green color represents non-coding regions (5′ untranslated region (UTR) and 3′ UTR).

To compare mutation patterns across subtypes, mutation frequencies were averaged at each site within each subtype. Sites missing more than two-thirds of the total sample size for the subtype were excluded, which resulted in a data table for each subtype with the numbers of usable sites of 8312 (subtype 1a), 8282 (subtype 1b), and 8349 (subtype 3a) sites between the genome positions 264 to 8649 (Figure 1).

The coding region starts at the position 342, and therefore, for the analysis involving types of mutations (synonymous/nonsynonymous/nonsense), the first 78 sites that are part of the 5′ UTR were excluded. Average mutation frequencies were calculated for each subtype across the polyprotein coding region, as well as within each genomic region. We estimated within-host nucleotide diversity (π) using a method developed specifically for calculating a within-host nucleotide diversity of virus populations from NGS data [16] Comparison of frequencies and nucleotide diversity between different subtypes, genomic regions, and types of mutations was performed using Wilcoxon rank sum tests. Beta regression was conducted to understand the effects of different factors on mutation frequencies using the *betareg* package [30] in R. Predictor variables included ancestral nucleotide, mutation types (synonymous vs. nonsynonymous, CpG site-creating or not, drastic amino acid-changing or not), location (11 genomic regions), and interactions. The Akaike information criterion (AIC) values were used as a criterion to select the best-fit model.

In order to compare within-host (in vivo) evolutionary patterns to between-host patterns, we downloaded additional genome sequences from the NCBI GenBank. Each downloaded sequence represents a consensus sequence of a viral population (i.e., patient), and these sequences were used to estimate between-host evolutionary patterns. A total of 433 complete genome sequences for subtype 1a, 247 for subtype 1b, and 501 for subtype 3a were obtained, and the ratio of nonsynonymous to synonymous nucleotide diversity (π_N_/π_S_) was estimated by obtaining the standard pairwise nucleotide diversity using the Pegas package [31] in R. We estimated the values of the fixation index (F_ST_) at each nucleotide site, which was calculated as (π_total_-π_within-subtype_)_/_π_total_.

Known RAVs were compiled from existing publications [6,32,33,34,35,36] (Supplementary Dataset S2), and the prevalence of pre-existing RAVs in treatment-naïve participants (i.e., natural prevalence) was assessed for each subtype.

## 3. Results

### 3.1. Comparison of Within-Patient Evolutionary Patterns between HCV Subtypes

#### 3.1.1. Within-Host Mutation Frequency Summary

**Figure 2:**
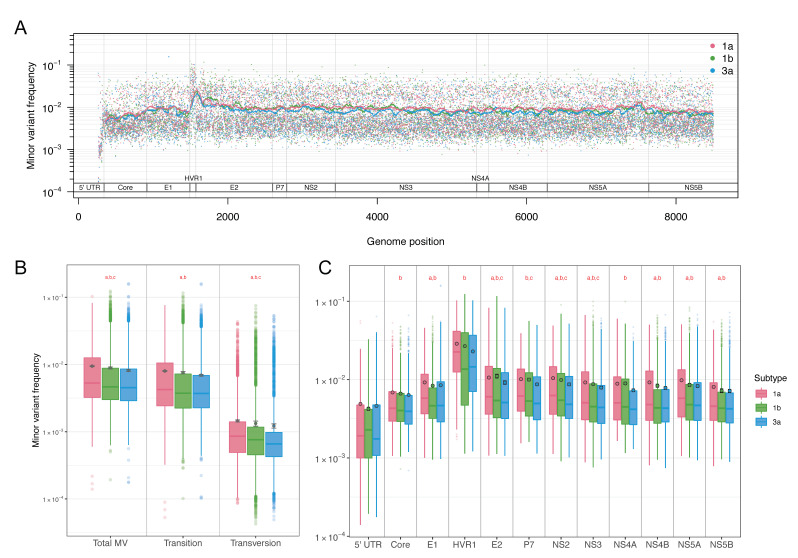
Within-host minor variant frequencies (MVFs) of HCV subtype 1a, 1b, and 3a. (**A**) MVFs across the genome with a 100-base rolling average, (**B**) a box plot of MVFs for total, transition, and transversion mutations, and (**C**) a box plot of MVFs per genomic region. Circles in (**B**,**C**) represent the means with standard errors. Letters in (**B**,**C**) represent statistically significant differences between subtype 1a and 1b (a), subtype 1a and subtype 3a (b), and subtype 1b and subtype 3a (c).

Comparative analyses of genomes from three HCV subtypes were conducted using the NGS data from treatment-naive CCC participants (subtype 1a: *n* = 195, 1b: *n* = 21, and 3a: *n* = 39). The sample size reflects the overall prevalence of HCV subtypes in the human population, with subtype 1a being the dominant subtype worldwide [37]. MVFs at each nucleotide site were estimated by averaging MVFs across samples within each subtype. The MVFs along the genome of all subtypes showed a similar overall pattern, with a clear peak in HVR1 (Figure 2A). The average MVFs observed were just below 1% (8.15–9.45 (±0.11–0.29) × 10^−3^ per nucleotide), of which the average MVFs resulting from transition mutations were just below 1% (6.92–8.00 (±0.10–0.27) × 10^−3^), and the average MVFs resulting from transversion mutations were just over 0.1% (1.22–1.45 (±0.04–0.11) × 10^−3^) (Figure 2B, Table S1). In all three categories, subtype 1a had the highest average MVFs, and subtype 3a had the lowest. The differences in the average MVFs were all statistically significant (Wilcoxon rank-sum test, *p* < 0.0001), except for the transition mutation frequencies between subtype 1b and subtype 3a (*p* = 0.16), which may be due to the smaller sample sizes for these subtypes.

For some of our analyses, we split the HCV genome into 12 parts or regions (the 5′ UTR, 10 genes, and HVR1, which is part of gene E2). Subtype 1a had the highest MVF averages in 10 out of 12 regions, and subtype 3a had the lowest averages in 10 out of 12 genes (Figure 2C). Statistically, subtype 1a had significantly higher MVFs in seven regions than subtype 1b, and in 11 regions than subtype 3a (Wilcoxon test, *p*-values range from 4.05 × 10^−9^ to 0.048). Since transition mutations occur at much higher frequencies, our analysis hereafter focuses on transition mutations to gain more statistical power and accuracy (i.e., MVF hereafter refers to ‘minor transition variant frequency’).

#### 3.1.2. Nonsynonymous and Synonymous Transition Mutation Frequencies by Genomic Region

At each site, there is only one possible transition mutation. This mutation is a synonymous, a non-synonymous, or a nonsense (stop) mutation. We split all mutations in the 11 genomic regions (excluding 5′ UTR) into synonymous, non-synonymous, and nonsense mutations. The average transition MVFs and nucleotide diversity (π) were compared between subtypes. For this analysis, sites that did not have the same nucleotide as the reference sequence were excluded from each sample.

**Figure 3:**
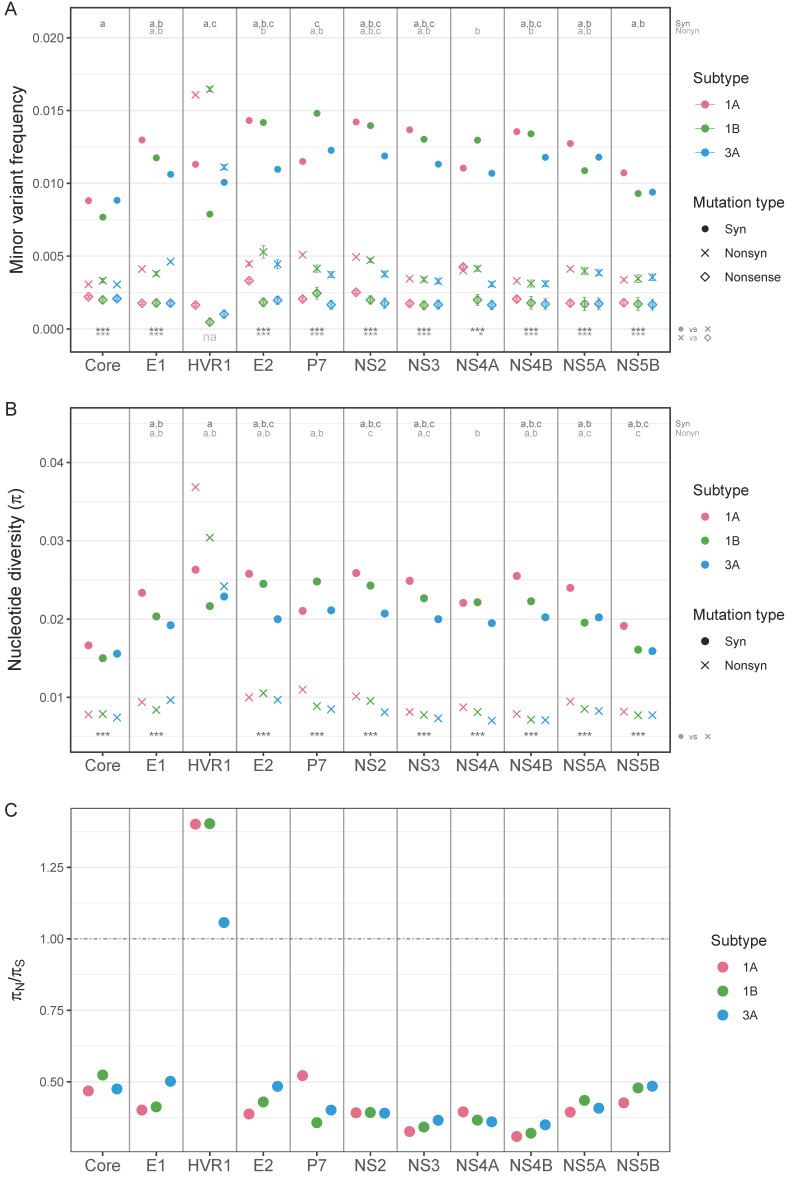
Within-host MVFs of HCV subtype 1a, 1b, and 3a. (**A**) MVFs across the genome with a 100-base rolling average, (**B**) a box plot of MVFs for total, transition, and transversion mutations, and (**C**) a box plot of MVFs per genomic region. Circles in (**B**,**C**) represent the means with standard errors. Letters on the top in (**A**,**B**) represent statistically significant differences between subtype 1a and 1b (letter ‘a’), subtype 1a and subtype 3a (letter ‘b’), and subtype 1b and subtype 3a (letter ‘c’). Asterisks (*) on the bottom in (**A**,**B**) represent a statistically significant difference between mutation types (Synonymous vs. nonsynonymous, nonsynonymous vs. nonsense) within each subtype (1a, 1b and 3a from the left).

First, we compared MVFs and nucleotide diversity of different mutation types per region within each subtype. The results revealed that, except for HVR1, the average MVFs were significantly lower at nonsynonymous sites than at synonymous sites in all regions in all subtypes (Wilcoxon test, *p*-values range from 1.65 × 10^−228^ to 8.93 × 10^−15^, Figure 3A). HVR1 had higher average MVFs at nonsynonymous sites than at synonymous sites, though the differences were not statistically significant. The average MVFs at nonsense sites were also compared to those at nonsynonymous sites. Nonsense sites had significantly lower MVFs than nonsynonymous sites in all regions in all subtypes (Wilcoxon test, *p*-values range from 3.97 × 10^−10^ to 1.95 × 10^−15^), except for HVR1 and P7. Here, HVR1 was excluded from statistical analysis due to the small sample sizes (HVR1 had only 1 or 2 nonsense mutations per subtype). For P7, only subtype 3a had significantly lower MVFs at nonsense sites than at nonsynonymous sites (Wilcoxon test, *p* = 0.019). Comparison of nucleotide diversity between synonymous and nonsynonymous sites showed the same pattern as MVFs; all but HVR1 had significantly lower nucleotide diversity at nonsynonymous sites than at synonymous sites in all subtypes (Wilcoxon test, *p*-values range from 1.80 × 10^−209^ to 4.68 × 10^−14^, Figure 3B). For HVR1, nucleotide diversity was higher at nonsynonymous sites than at synonymous sites in all subtypes, but the difference was again not statistically significant.

Second, we compared MVFs and nucleotide diversity between subtypes per region, which followed the overall pattern of subtype 1a having the highest values, and subtype 3a having the lowest values in most regions. For synonymous mutations, subtype 1a had the highest MVFs in seven out of 11 regions (Wilcoxon test, *p*-values range from 2.2 × 10^−16^ to 0.003), while subtype 3a had the lowest average MVFs in 9 regions, 4 of which were statistically significant (Wilcoxon test, *p*-values range from 0.009–0.02, Figure 3A). Differences between the subtypes were less pronounced for nonsynonymous mutations: subtype 1a had significantly higher nonsynonymous MVFs in five regions than subtype 1b and subtype 3a (Wilcoxon test, *p*-values range from 0.001 to 0.02), but none of the regions in subtype 3a had significantly lower or higher MVFs than those of subtype 1b. Similarly, nucleotide diversity was the highest in subtype 1a in the majority of regions: at synonymous sites, subtype 1a had a significantly higher diversity in eight (vs. 1b) and nine (vs. 3a) regions, and at nonsynonymous sites, in six (vs. 1b) and eight (vs. 3a) regions (Figure 3B). The comparison between subtype 1b and subtype 3a resulted in fewer regions being significantly different (four at synonymous sites, and two at nonsynonymous sites).

#### 3.1.3. Sites Conserved across Different Subtypes Have Lower Mutation Frequencies

**Figure 4:**
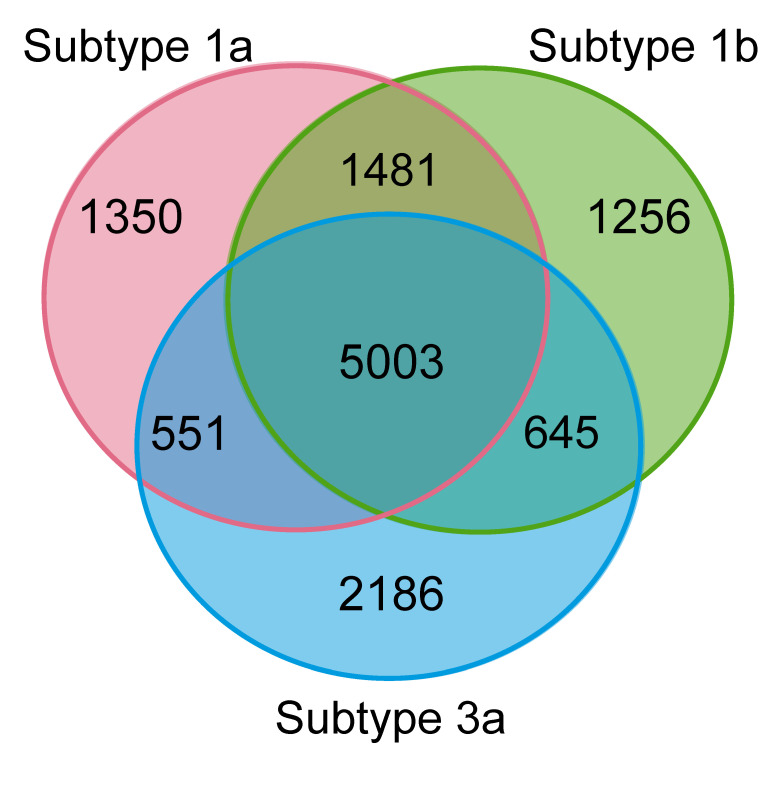
Venn diagram showing the number of sites that were ‘conserved’ (have the same nucleotide and mutation type) between subtypes.

**Figure 5:**
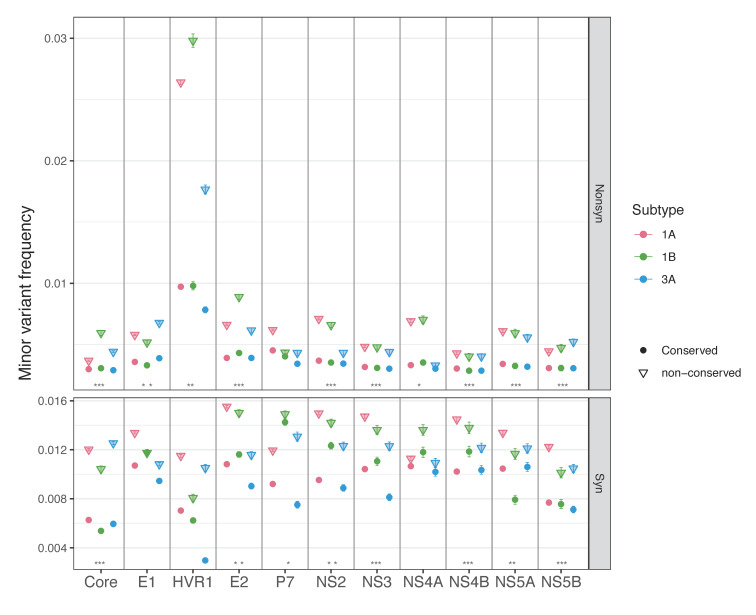
Comparison of average MVFs between ‘conserved sites’ and the rest of the sites (‘non-conserved sites’). The average MVFs were plotted separately for synonymous (Syn) mutations and nonsynonymous (Nonsyn) mutations. Error bars denote standard errors. Asterisks (*) denote a statistically significant difference between conserved and non-conserved sites for each subtype (from left, subtype 1a, 1b, and 3a).

To obtain further insights into the differences of within-host evolutionary patterns among subtypes, we compared MVFs and nucleotide diversity for sites that have the same ancestral nucleotide and mutation type between subtypes (e.g., “A” that results in a “synonymous” change at the genome position 359 in both subtype 1a and subtype 1b; referred to as “conserved sites” hereafter). Pairwise comparison of conserved sites between the two subtypes revealed that 77.3% of the studied sites were conserved sites between subtype 1a and subtype 1b (5003 + 1481 sites), 66.2% between subtype 1a and subtype 3a (5003 + 551 sites), and 67.4% between subtype 1b and subtype 3a (5003 + 645 sites). The proportion of conserved sites across the three subtypes was 59.7% (5003 sites) (Figure 4), of which 81.4% were nonsynonymous (4072 sites, which is 49.0% of the studied sites). The average MVFs at conserved sites were compared to those of non-conserved sites, which revealed that MVFs were significantly lower at conserved sites in nonsynonymous sites for the majority of genomic regions (Wilcoxon rank-sum test, *p*-values range from 3.64 × 10^−13^ to 0.029, Figure 5), while for synonymous sites, about a half of the comparisons were significantly lower for conserved sites (Wilcoxon rank-sum test, *p*-values range from 6.85 × 10^−10^–0.023).

Site-by-site comparison of MVFs between subtypes at conserved sites also showed 2.6–4.3-time higher correlation (Spearman’s correlation, rho-value range 0.68–0.76) than at non-conserved sites (Spearman’s correlation, rho-values 0.18–0.26, Table S2). However, all correlations were significant even for non-conserved sites (*p* < 4.5 × 10^−11^), and this genome-wide high correlation of MVFs across subtypes indicates overall selective pressures or evolutionary constraints are similar across subtypes.

We also estimated the values of the fixation index (F_ST_) between subtypes, using the site-by-site F_ST_ values averaging across the genome, which resulted in over 50% higher values between different genotypes (1a vs. 3a: *F_ST_* = 0.307 and 1b vs. 3a: 0.299) than within a genotype (1a vs. 1b: *F_ST_* = 0.199). The aggregate nucleotide diversity values within each subtype and between subtypes also followed a similar pattern (Figure S2).

### 3.2. What Drives and Constrains Within-Host Mutations?

Multiple factors simultaneously drive and constrain mutation frequencies, and these effects were explored using beta-regression models to understand the driving force of similarities and differences in mutation patterns across subtypes. We explored the effects of ancestral nucleotide, mutation type (synonymous/nonsynonymous, whether a mutation creates a CpG-site or not or causes a drastic amino acid (AA) change or not), location in the genome, and interactions of these factors, as they were known to shape mutation patterns in HCV [19]. The best-fit model included a total of 15 factors (Table S3). In our model, the effect size of each factor on mutation frequencies was estimated from the baseline, which was a ‘synonymous, non-CpG, non-drastic AA change mutation at nucleotide A’.

**Figure 6:**
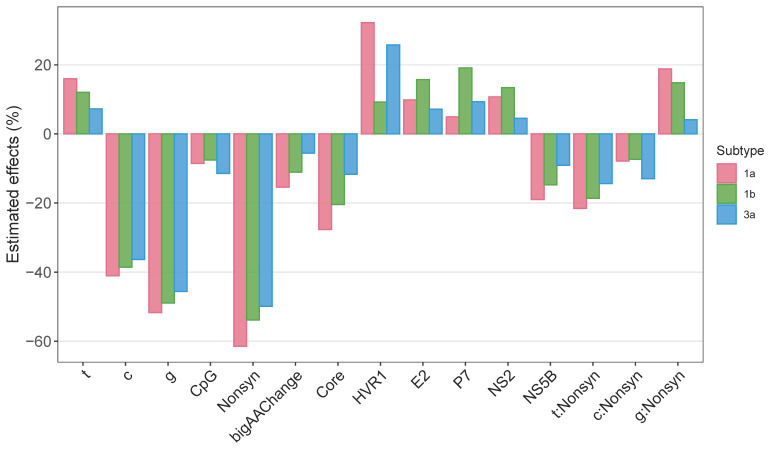
Effects of various factors on mutation frequencies estimated using beta regression. Predicted effect sizes of ancestral nucleotide (t, c, or g vs. a); CpG-creating status (CpG; vs. non-CpG-creating status); nonsynonymous (Nonsyn; vs. synonymous); drastic amino acid-changing status (AAChange; vs. non-drastic amino acid-changing); genomic locations (Core, HVR1, E2, P7, NS2, NS4A, and NS5B; vs. the NS3/NS4B/NS5A regions) on mutation frequencies in the HCV genome. The figure reflects the result of the best-fit model based on AIC from the beta regression.

The directions of the effects (i.e., to increase or decrease mutation frequencies) were the same across all three subtypes (Figure 6). The overall magnitudes of the effects were also comparable across the three subtypes: the predicted mutation frequencies from the best-fit model did not differ significantly among subtypes (χ^2^-test, *p* = 1). Whether a mutation was nonsynonymous or not had the largest negative effect on mutation frequencies (−48.6 to −61.4%), while HVR1 had the largest positive effect (22.2 to 30.3 %). Subtype 1a had the largest effect size in the majority of factors (10 out of 15), while subtype 3a had the smallest effect size in the majority of factors (11 out of 15). However, the effect of CpG-creating mutations was largest in subtype 3a (−11.3 % in 3a, −8.5% in 1a, −6.0% in 1b), and the effects of locations also showed variable results between subtypes.

### 3.3. Resistance-Associated Variants (RAVs)

**Figure 7:**
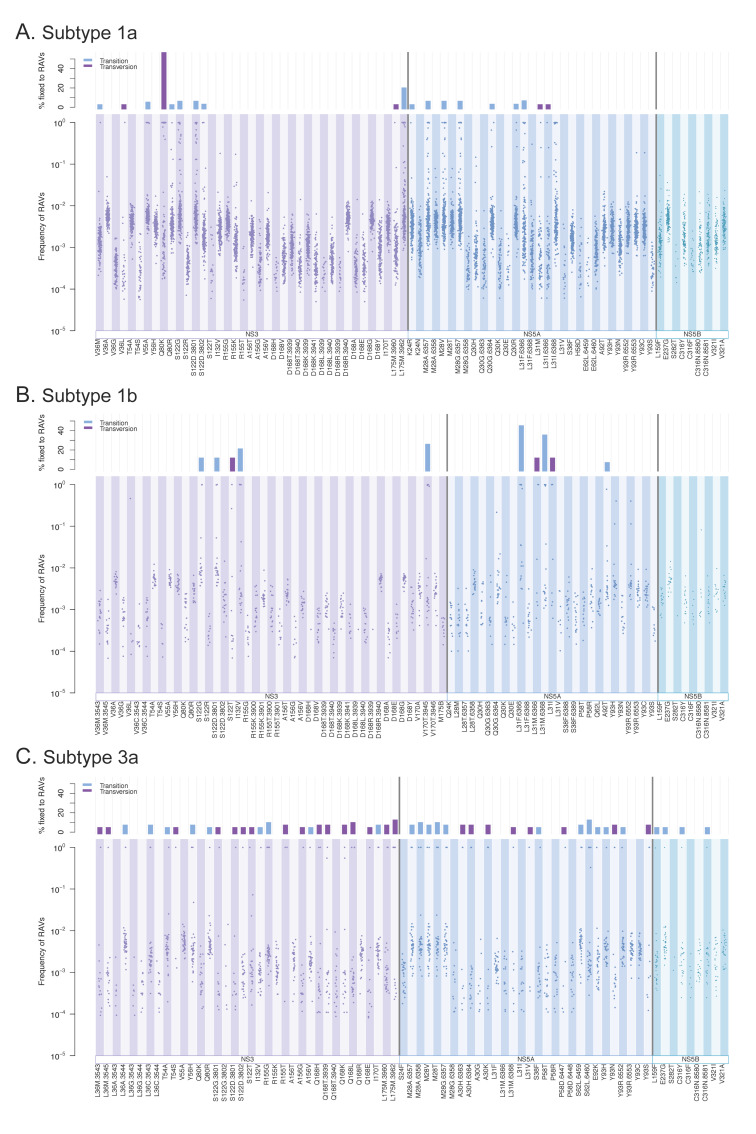
Natural occurrence of resistance-associated variants (RAVs) in HCV subtypes ((**A**): subtype 1a, (**B**): subtype 1b, (**C**): subtype 3a). The upper plot in each figure shows the proportion (%) of samples with RAVs as a majority nucleotide within each subtype. The bottom figure shows the observed frequency of RAVs. Each dot represents a RAV frequency at a specified location in each sample (patient).

The natural occurrence of known RAVs was explored for each subtype, which revealed the consistent presence of RAVs in treatment-naïve participants (Figure 7). Direct-acting antiviral drugs that target the NS3, NS5A, or NS5B proteins have been developed for HCV [6]. A number of RAVs developed in patients have been reported, of which we assessed 79 variants for subtype 1a, 81 for subtype 1b, and 75 for subtype 3a that spanned over 27 sites (Supplemental Dataset S2). We observed all RAVs in at least several samples, with average percentages of RAVs ranging from 58.5 to 72.8% (Table S4A). Most RAVs were observed at low frequencies (average 0.0017–0.0032 for non-fixed RAVs, Table S4A), but certain RAVs were found to be fixed in some samples (i.e., an RAV as a majority nucleotide in the viral population). The proportion of RAVs that were observed to be fixed in at least one sample was relatively low for subtype 1a (*n* = 20 RAVs, 25.3%) and 1b (*n* = 10 RAVs, 12.3%), but high for subtype 3a (*n* = 48 RAVs, 64.0%). The average observed proportion of fixed samples for each RAV was low for subtype 1a (5.66%) and subtype 3a (4.30%), except for Q80K of NS3A in subtype 1a (54.4%), which is known to be prevalent in patients from North America [34]. The average proportion of fixed samples per RAV was higher for subtype 1b (17.1%), but this may be due to its small sample size (*n* = 21 patient samples). The highest fixed RAV proportion in subtype 1b (42.9%) was seen at L31F of NS5A (nucleotide position = 6366).

Comparing the occurrence of RAVs among three genes revealed that the proportions of samples with RAVs among genes differed between subtypes 3a and subtypes 1a/1b. In subtypes 1a/1b, NS5B had the lowest proportion of samples with RAVs (54–63%), while NS3 had the highest (70–74%). In contrast, in subtype 3a, the observed proportions were similar among three genes (57.5–59%, Table S4B). Our datasets included limited regions of NS5B and thus assessed only nine RAVs from NS5B. However, interestingly, fixed RAVs in this gene were observed only in subtype 3a (*n* = 4 RAVs, average 2.56% of samples per RAV, Table S4B).

### 3.4. Between- vs. within-Host Genetic Variation

**Figure 8:**
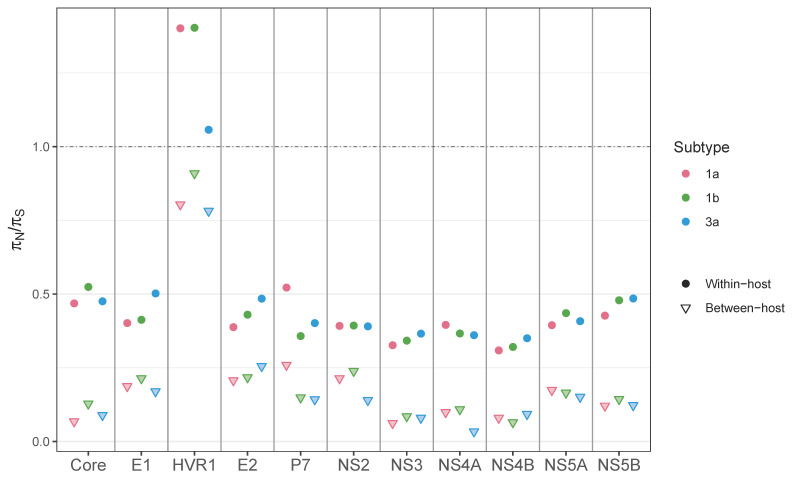
Between-host selective pressures (πN_/_/πS) on different HCV genomic regions, in comparison with those within hosts (in vivo).

To understand how selective pressures act differently across different scales, we compared the ratios of nucleotide diversity (π_N_/π_S_) within hosts and between hosts. A π_N_/π_S_ value of 1 suggests neutrality or absence of selective pressure, whereas a value > 1 suggests the presence of positive (diversifying) selection, and a value < 1 suggests negative (purifying) selection. Nucleotide diversity between hosts was calculated from full genome sequences obtained from NCBI, combined with consensus sequences from our dataset (Figure S3). The πN/πS ratios calculated based on genomic regions revealed the value of HVR1 to be >1 within hosts, indicating HVR1 being under positive or diversifying selection, consistent with previous findings [13,18] (Figure 8). The rest of the genomic regions appeared to be under purifying selection, with a comparable degree of selective pressures among subtypes. The πN/πS ratios between hosts followed a similar overall pattern to those within hosts, with HVR1 having prominently higher values than the rest of the regions. However, the between-host πN/πS ratios at HVR1 were <1 in all three subtypes, indicating that the selective pressure at HVR1 between hosts was slightly negative, though it was much more relaxed than in the rest of the regions. Also, in all genomic regions, the between-host πN/πS ratios were much lower than those within hosts. The results illustrated that the evolutionary pattern between hosts was driven by stronger purifying selection than within hosts, and many nonsynonymous mutations that accumulate within hosts are removed during the viral transmission process. Indeed, HCV infections are reported to start with one or very few variants in most patients [38], suggesting that the evolutionary force at the bottleneck transmission event is different from that within host.

## 4. Discussion

In this study, we quantitatively assessed within-host mutation patterns across three different subtypes of HCV using a large number of NGS samples to gain much-needed insights into within-host, in vivo molecular evolution. We showed that NGS generates ideal data to study within-host mutational patterns of viral populations. They allow us to investigate mutations that occur at a low frequency in a viral population that traditional sequencing methods or consensus-level sequences cannot capture. Previous studies on genetic diversity of HCV were conducted primarily at the between-host level. With NGS technology, which has become more accessible and affordable over the last decades, we can gain a more rigorous understanding of the complex within-host evolutionary patterns and diversity of natural viral populations.

Our study confirmed that results from our previous study on subtype 1a [19] generally also hold for subtypes 1b and 3a. Genetic diversity is the lowest in the 5′ UTR (the 3′ UTR was not included in our study) and, by far, the highest in the Hyper Variable Region 1 (HVR1), which is part of the E2 gene. A beta regression analysis found that in all three subtypes, mutation frequencies were also somewhat higher in the genes neighboring HVR1 on the 3′ side (E2, P7, and NS2), while somewhat lower on the edges of the genome (the Core gene right next to the 5′ UTR and the NS5B gene, next to the 3′ UTR, see Figure 6). Throughout the genome, mutation frequencies were reduced by ≧50% for nonsynonymous mutations (as expected), and there was a small effect of whether mutations created CpG dinucleotides, which reduced mutation frequencies by 7.5–11.4% (*p*-value = 4.6 × 10^−9^ − 0.0002, Figure 6, Table S3). CpG dinucleotides are underrepresented, and CpG-creating mutations are costly in many human viruses [39,40,41]. In HCV, removal of CpG dinucleotides enhanced the replication of subtypes 1b and 2a in vitro [42]. Our study, therefore, confirms that CpG-creating mutations in HCV also come with a statistically significant, yet small fitness cost compared to other human viruses [41].

In addition to clear similarities, we also found significant differences between the three subtypes in genome-wide mutation frequencies, as well as consistent differences in the degree of genetic variability: subtype 3a had the lowest mutation frequencies and genetic diversity levels (Figure 2 and Figure 3), while subtype 1a had the highest. This suggests that different subtypes have inherent differences in population-level evolutionary dynamics. Subtype 1a is most variable within patients and also the most common subtype globally. It is not clear if these two observations are connected. Although our samples did not show differences in viral loads between subtypes, genotype 1 has been reported to have higher viral loads than genotype 3 [43,44,45]. The viral load of HCV is also reported to be associated with certain host genotypes and a specific amino acid of the viral NS5A protein [46]. It appears that the relationship between genetic variability, viral load, and transmission effectiveness in HCV is complex, and its understanding requires further research.

Despite the HCV’s high level of mutagenesis, our results show that approximately 50% of genomic sites are evolutionarily conserved across the three genotypes, subject to strong purifying selection. Within-host mutation frequencies were lower at these conserved sites than at non-conserved sites (Figure 5). This suggests that these sites are under purifying selection, both at the within-host and at the between-host level in all three HCV subtypes we studied.

When we compared the two subtypes within genotype 1, within-host mutation frequencies and nucleotide diversity were clearly higher for subtype 1a than for subtype 1b in the majority of regions. The results agree with previous findings [13], which reported a higher between-host evolutionary rate for subtype 1a than for subtype 1b. Gray et al. [13] also found that the codon rate ratios (the evolutionary rate at codon positions 1 and 2 compared to that at codon position 3), which correlate with dN/dS ratios and can be interpreted in the same way as *dN*/*dS* or πN/πS, exceeded 1 only in the region containing HVR1 within hosts, but not between hosts. The concurrent results we obtained (Figure 8) suggest that the HVR1 region goes through strong immune-mediated positive or diversifying selection within a patient, but a substantial portion of nonsynonymous mutations are removed during transmission. Xue et al. [47] reported similar findings that nonsynonymous mutations are detectably purged between hosts, while they accumulate at a comparable rate to that of synonymous mutations within hosts during acute influenza virus infection.

In our study, subtype 3a had the lowest genetic variation, and subtype 1a the highest. This could lead us to predict that drug resistance evolution may be more likely for subtype 1a. Data on treatment outcomes do not show this pattern. Instead, genotype 3 is generally reported to show poorer responses to antiviral drug treatments than genotype 1 (e.g., [48,49]). This treatment difficulty may be related to the higher occurrence of RAVs in treatment-naïve patients. Our results on RAVs revealed that subtype 3a had the highest natural prevalence of RAVs that were fixed: 65% of RAVs examined in subtype 3a had one or more samples with RAVs as majority nucleotides (Figure 5).

In summary, analyzing within-host viral diversity and variability, especially low-frequency variants, deepens our understanding of the evolutionary dynamics of HCV, which, as we showed in this study, are quite complex and distinct from between-host dynamics. With the cost of NGS becoming increasingly more affordable, we have tremendous opportunities to expand our knowledge on within-host evolution of disease viruses. For clinical application, this can help us predict mutational effects on the regions of interest within a viral genome and identify potential candidates of RAVs for new drugs, all of which will contribute to improving existing treatment, as well as developing new treatments.

## Data Availability

Processed bam files are available at FigShare (https://figshare.com/s/f31f4221421d6923c1ee, accessed on 16 November 2020). Scripts to analyze data are available at GitHub (http://github.com/kahot/HCV_mutation_patterns, accessed on 29 January 2021).

## Supplementary Materials

The following are available online at https://www.mdpi.com/1999-4915/13/3/511/s1, Figure S1: Relationship between viral load and nucleotide diversity (π) or average read depth of all HCV samples used in our analyses, Figure S2: Nucleotide diversity (π) and fixation indices (F_ST_) between HCV subtypes, Figure S3: Between-host nucleotide diversity (π) of each subtype, Table S1: Estimated average minor variant frequencies based on HCV subtypes, Table S2: Results of beta regression, showing the effects of different factors affecting mutation frequencies for each subtype, Table S3: Spearman’s correlation coefficient (rho) values of MVFs at conserved sites between two subtypes and those at non-conserved sites between two subtypes, Table S4: Proportions and frequencies of observed known resistance associated variants (RAVs) assessed in our study, Supplementary Dataset S1: Read depth and viral load information of the samples, Supplementary Dataset S2: Resistant-associated variants investigated in our study.

## References

[1] Kato N. (2000). Genome of Human Hepatitis C Virus (HCV): Gene organization, sequence diversity, and variation. Microb. Comp. Genom..

[2] Jefferies M., Rauff B., Rashid H., Lam T., Rafiq S. (2018). Update on global epidemiology of viral hepatitis and preventive strategies. WJCC.

[3] Coppola N., Alessio L., Onorato L., Sagnelli C., Macera M., Sagnelli E., Pisaturo M. (2019). Epidemiology and management of hepatitis C virus infections in immigrant populations. Infect. Dis. Poverty.

[4] Lauring A.S., Andino R. (2010). Quasispecies Theory and the Behavior of RNA Viruses. PLoS Pathog..

[5] Gallego I., Gregori J., Soria M.E., García-Crespo C., García-Álvarez M., Gómez-González A., Valiergue R., Gómez J., Esteban J.I., Quer J. (2018). Resistance of high fitness hepatitis C virus to lethal mutagenesis. Virology.

[6] Sorbo M.C., Cento V., Di Maio V.C., Howe A.Y.M., Garcia F., Perno C.F., Ceccherini-Silberstein F. (2018). Hepatitis C virus drug resistance associated substitutions and their clinical relevance: Update 2018. Drug Resist. Updates.

[7] WHO Hepatitis C. https://www.who.int/news-room/fact-sheets/detail/hepatitis-c.

[8] Echeverría N. (2015). Hepatitis C virus genetic variability and evolution. WJH.

[9] Mauger D.M., Golden M., Yamane D., Williford S., Lemon S.M., Martin D.P., Weeks K.M. (2015). Functionally conserved architecture of hepatitis C virus RNA genomes. Proc. Natl. Acad. Sci. USA.

[10] Smith D.B., Pathirana S., Davidson F., Lawlor E., Power J., Yap P.L., Simmonds P. (1997). The origin of hepatitis C virus genotypes. J. Gen. Virol..

[11] Major M.E., Feinstone S.M. (1997). The molecular virology of hepatitis C. Hepatology.

[12] Keck Z.-Y., Girard-Blanc C., Wang W., Lau P., Zuiani A., Rey F.A., Krey T., Diamond M.S., Foung S.K.H. (2016). Antibody Response to Hypervariable Region 1 Interferes with Broadly Neutralizing Antibodies to Hepatitis C Virus. J. Virol..

[13] Gray R.R., Parker J., Lemey P., Salemi M., Katzourakis A., Pybus O.G. (2011). The mode and tempo of hepatitis C virus evolution within and among hosts. BMC Evol. Biol..

[14] Di Lello F.A., Culasso A.C.A., Campos R.H. (2015). Inter and intrapatient evolution of hepatitis C virus. Ann. Hepatol..

[15] Alizon S., Luciani F., Regoes R.R. (2011). Epidemiological and clinical consequences of within-host evolution. Trends Microbiol..

[16] Nelson C.W., Hughes A.L. (2015). Within-host nucleotide diversity of virus populations: Insights from next-generation sequencing. Infect. Genet. Evol..

[17] Caporossi A., Kulkarni O., Blum M.G.B., Leroy V., Morand P., Larrat S., François O. (2019). Using high-throughput sequencing for investigating intra-host hepatitis C evolution over long retrospective periods. Infect. Genet. Evol..

[18] Raghwani J., Wu C.-H., Ho C.K.Y., de Jong M., Molenkamp R., Schinkel J., Pybus O.G., Lythgoe K.A. (2019). High-Resolution Evolutionary Analysis of Within-Host Hepatitis C Virus Infection. J. Infect. Dis..

[19] Tisthammer K.H., Dong W., Joy J.B., Pennings P.S. (2020). Assessing in vivo mutation frequencies and creating a high-resolution genome-wide map of fitness costs of Hepatitis C virus. bioRxiv.

[20] Lindsay K.L. (2003). Introduction to therapy of hepatitis C. J. Med. Virol..

[21] Huang J.-F., Huang C.-F., Yeh M.-L., Dai C.-Y., Yu M.-L., Chuang W.-L. (2018). Updates in the management and treatment of HCV genotype 3, what are the remaining challenges?. Expert Rev. Anti-Infect. Ther..

[22] Klein M.B., Saeed S., Yang H., Cohen J., Conway B., Cooper C., Côté P., Cox J., Gill J., Haase D. (2010). Cohort profile: The Canadian HIV–hepatitis C co-infection cohort study. Int. J. Epidemiol..

[23] Bushnell B., Rood J., Singer E. (2017). BBMerge—Accurate paired shotgun read merging via overlap. PLoS ONE.

[24] Li H., Durbin R. (2009). Fast and accurate short read alignment with Burrows-Wheeler transform. Bioinformatics.

[25] Zanini F., Brodin J., Thebo L., Lanz C., Bratt G., Elife J.A., Neher R.A. (2015). Population genomics of intrapatient HIV-1 evolution. eLife.

[26] Li H. (2011). A statistical framework for SNP calling, mutation discovery, association mapping and population genetical parameter estimation from sequencing data. Bioinformatics.

[27] R Core Team (2020). R: A Language and Environment for Statistical Computing.

[28] Chui C.K.S., Dong W.W.Y., Joy J.B., Poon A.F.Y., Dong W.Y., Mo T., Woods C.K., Beatty C., Hew H., Harrigan P.R. (2015). Development and Validation of Two Screening Assays for the Hepatitis C Virus NS3 Q80K Polymorphism Associated with Reduced Response to Combination Treatment Regimens Containing Simeprevir. J. Clin. Microbiol..

[29] Fox E.J., Reid-Bayliss K.S., Emond M.J., Loeb L.A. (2014). Accuracy of Next Generation Sequencing Platforms. Next Gener. Seq. Appl..

[30] Caribari-Neto F., Zeileis A. (2019). Beta Regression in R. J. Stat. Softw..

[31] Paradis E., Jombart T., Kamvar Z., Knaus B., Sochliep K., Potts A., Winter D. (2010). Package “pegas”: An R package for population genetics with an integrated–modular approach. Bioinformatics..

[32] Poveda E., Wyles D.L., Mena Á., Pedreira J.D., Castro-Iglesias Á., Cachay E. (2014). Update on hepatitis C virus resistance to direct-acting antiviral agents. Antiviral Res..

[33] Lontok E., Harrington P., Howe A., Kieffer T., Lennerstrand J., Lenz O., McPhee F., Mo H., Parkin N., Pilot-Matias T. (2015). Hepatitis C virus drug resistance-associated substitutions: State of the art summary. Hepatology.

[34] Sarrazin C. (2016). The importance of resistance to direct antiviral drugs in HCV infection in clinical practice. J. Hepatol..

[35] Kim S., Han K.-H., Ahn S.H. (2016). Hepatitis C Virus and Antiviral Drug Resistance. Gut Liver.

[36] Wyles D.L., Luetkemeyer A.F. (2017). Understanding Hepatitis C Virus Drug Resistance: Clinical Implications for Current and Future Regimens. Top. Antivir. Med..

[37] Petruzziello A., Marigliano S., Loquercio G., Cozzolino A., Cacciapuoti C. (2016). Global epidemiology of hepatitis C virus infection: An up-date of the distribution and circulation of hepatitis C virus genotypes. World J. Gastroenterol..

[38] Abayasingam A., Leung P., Eltahla A., Bull R.A., Luciani F., Grebely J., Dore G.J., Applegate T., Page K., Bruneau J. (2019). Genomic characterization of hepatitis C virus transmitted founder variants with deep sequencing. Infect. Genet. Evol..

[39] Karlin S., Doerfler W., Cardon L.R. (1994). Why is CpG suppressed in the genomes of virtually all small eukaryotic viruses but not in those of large eukaryotic viruses?. J. Virol..

[40] Cheng X., Virk N., Chen W., Ji S., Ji S., Sun Y., Wu X. (2013). CpG usage in RNA viruses: Data and hypotheses. PLoS ONE.

[41] Caudill V.R., Qin S., Winstead R., Kaur J., Tisthammer K., Pineda E.G., Solis C., Cobey S., Bedford T., Carja O. (2020). CpG-creating mutations are costly in many human viruses. Evol. Ecol..

[42] Witteveldt J., Martin-Gans M., Simmonds P. (2016). Enhancement of the Replication of Hepatitis C Virus Replicons of Genotypes 1 to 4 by Manipulation of CpG and UpA Dinucleotide Frequencies and Use of Cell Lines Expressing SECL14L2 for Antiviral Resistance Testing. Antimicrob. Agents Chemother..

[43] Berger A., Prondzinski M.D., Doerr H.W., Rabenau H., Weber B. (1996). Hepatitis C plasma viral load is associated with HCV genotype but not with HIV coinfection. Conserv. Lett..

[44] Chakravarti A., Dogra G., Verma V., Srivastava A.P. (2011). Distribution pattern of HCV genotypes & its association with viral load. Indian J. Med. Res..

[45] Petruzziello A., Coppola N., Loquercio G., Marigliano S., Giordano M., Azzaro R., Diodato A.M., Iervolino V., Di Costanzo G., Di Macchia C.A. (2014). Distribution Pattern of Hepatitis C Virus Genotypes and Correlation with Viral Load and Risk Factors in Chronic Positive Patients. Intervirology.

[46] Ansari M.A., Pedergnana V., Ip C.L.C., Magri A., Von Delft A., Bonsall D., Chaturvedi N., Bartha I., Smith D., Nicholson G. (2017). Genome-to-genome analysis highlights the effect of the human innate and adaptive immune systems on the hepatitis C virus. Nat. Genet..

[47] Xue K.S., Bloom J.D. (2020). Linking influenza virus evolution within and between human hosts. Virus Evol..

[48] Ampuero J., Romero-Gómez M., Reddy K.R. (2014). Review article: HCV genotype 3—The new treatment challenge. Aliment. Pharmacol. Ther..

[49] Liu R., Curry S., McMonagle P., Yeh W.W., Ludmerer S.W., Jumes P.A., Marshall W.L., Kong S., Ingravallo P., Black S. (2015). Susceptibilities of Genotype 1a, 1b, and 3 Hepatitis C Virus Variants to the NS5A Inhibitor Elbasvir. Antimicrob. Agents Chemother..

