# Supplementary material for: Comparative Analysis of Within-Host Mutation Patterns and Diversity of Hepatitis C Virus Subtypes 1a, 1b, and 3a
Source: Viruses. 2021 Mar 19;13(3):511. doi: 10.3390/v13030511 (PMC8003410; doi:10.3390/v13030511)

Supplementary materials for:
Comparative analysis of within-host mutation patterns and diversity of Hepatitis C virus subtypes 1a, 1b, and 3a

Table S1. Estimated average minor variant frequencies based on HCV subtypes. Numbers shown are mean ± standard errors.

Subtype	Total MVF	Mutation Type<br>Transition	Transversion
1a (195)	$9.45 \pm 0.11 \times 10^{-3}$	$8.00 \pm 0.10 \times 10^{-3}$	$1.45 \pm 0.044 \times 10^{-3}$
1b (21)	$8.89 \pm 0.29 \times 10^{-3}$	$7.56 \pm 0.27 \times 10^{-3}$	$1.33 \pm 0.116 \times 10^{-3}$
3a (39)	$8.15 \pm 0.22 \times 10^{-3}$	$6.92 \pm 0.20 \times 10^{-3}$	$1.22 \pm 0.085 \times 10^{-3}$

Table S2. Spearman's correlation coefficient (rho) values of MVFs at conserved sites between two subtypes, and those at non-conserved sites between two subtypes.

Subtype	Correlation coefficient (rho) Conserved sites	Correlation coefficient (rho) Non-conserved sites
1a -1b	0.764***	0.176***
1a -3a	0.691***	0.258***
1b-3a	0.684***	0.205***

Table S3. Results of beta regression, showing the effects of different factors affecting mutation frequencies for each subtype. Factors assessed are ancestral nucleotide, genomic locations, and mutation types, including CpG-creating (CpG), drastic amino acid changing (bigAAChange), and synonymous/nonsynonymous (Nonsyn) mutations. Estimated mutation frequency (estMF) is based on the best fit model. The intercept represents a baseline, which is synonymous, non-CpG, no-drastic amino acid changing mutations at nucleotide A. The effect size (Effect) is calculated based on the baseline.

Factor	Estimate	Std Error	Z value	Pr(>|z|)	estMF	Effect (%)	Subtype
(Intercept)	-4.0367174	0.02341736	-172.38139	0	0.01765533	0.0%	1a
t	0.14800417	0.0266133	5.5612869	**2.68E-08**	0.02047167	16.0%	1a
c	-0.5278209	0.0262551	-20.103559	**6.87E-90**	0.01041469	-41.0%	1a
g	-0.7271422	0.02931961	-24.80054	**8.85E-136**	0.00853261	-51.7%	1a
CpG	-0.08865	0.01790706	-4.9505631	**7.40E-07**	0.01615756	-8.5%	1a
Nonsyn	-0.9527843	0.02942211	-32.383278	**4.72E-230**	0.00680906	-61.4%	1a
bigAAChange	-0.1687512	0.01877512	-8.9880183	**2.52E-19**	0.0149138	-15.5%	1a
Core	-0.3233991	0.02612697	-12.37798	**3.44E-35**	0.0127769	-27.6%	1a
HVR1	0.26498314	0.05904097	4.48812293	**7.19E-06**	0.02301212	30.3%	1a
E2	0.09418781	0.01810092	5.20348284	**1.96E-07**	0.01939908	9.9%	1a
P7	0.04833342	0.03935023	1.22828806	**0.21933885**	0.01852963	5.0%	1a
NS2	0.10206406	0.02181559	4.67849193	**2.89E-06**	0.01955248	10.7%	1a
NS5B	-0.2108189	0.02014824	-10.463392	**1.27E-25**	0.01429942	-19.0%	1a
t:Nonsyn	-0.2435706	0.03537144	-6.8860805	**5.74E-12**	0.01383868	-21.6%	1a
c:Nonsyn	-0.0818241	0.03602471	-2.2713326	**0.02312685**	0.01626823	-7.9%	1a
g:Nonsyn	0.17236289	0.03714087	4.64078761	**3.47E-06**	0.02097646	18.8%	1a
(Intercept)	-4.2020913	0.02857185	-147.07101	**0**	0.01496425	0.0%	1b
t	0.11372751	0.03312158	3.43363802	**0.00059554**	0.01676665	12.0%	1b
c	-0.4870235	0.03240564	-15.028971	**4.74E-51**	0.00919482	-38.6%	1b
g	-0.6718044	0.03552149	-18.912616	**8.98E-80**	0.00764353	-48.9%	1b
CpG	-0.0782818	0.0213354	-3.6691039	**0.0002434**	0.0138375	-7.5%	1b
Nonsyn	-0.7726071	0.03501141	-22.067295	**6.52E-108**	0.0069106	-53.8%	1b
bigAAChange	-0.1170249	0.02151522	-5.4391685	**5.35E-08**	0.01331164	-11.0%	1b
Core	-0.2285458	0.03049961	-7.4934004	**6.71E-14**	0.0119069	-20.4%	1b
HVR1	0.0883893	0.08489916	1.04110913	**0.2978249**	0.01634715	9.2%	1b
E2	0.14619992	0.02145503	6.81425028	**9.48E-12**	0.01732003	15.7%	1b
P7	0.17488765	0.04463785	3.917923	**8.93E-05**	0.0178241	19.1%	1b
NS2	0.12594932	0.02582605	4.87683238	**1.08E-06**	0.01697282	13.4%	1b
NS5B	-0.1596108	0.0235227	-6.785397	**1.16E-11**	0.01275666	-14.8%	1b
t:Nonsyn	-0.2059795	0.04259487	-4.8357815	**1.33E-06**	0.01217865	-18.6%	1b
c:Nonsyn	-0.0764409	0.04222461	-1.8103408	**0.07024295**	0.013863	-7.4%	1b
g:Nonsyn	0.13804542	0.04361223	3.16529133	**0.00154928**	0.01717937	14.8%	1b
(Intercept)	-4.3628407	0.02578021	-169.23214	**0**	0.01274214	0.0%	3a
t	0.07013702	0.02995164	2.34167516	**0.01919742**	0.01366792	7.3%	3a
c	-0.4512945	0.03100733	-14.554449	**5.47E-48**	0.00811424	-36.3%	3a
g	-0.6082363	0.03493201	-17.412003	**6.69E-68**	0.00693567	-45.6%	3a
CpG	-0.1213156	0.02070036	-5.8605559	**4.61E-09**	0.01128641	-11.4%	3a
Nonsyn	-0.6906344	0.03311552	-20.855307	**1.36E-96**	0.0063871	-49.9%	3a
bigAAChange	-0.0574132	0.02144674	-2.6770133	**0.00742817**	0.01203118	-5.6%	3a
Core	-0.1243558	0.02958727	-4.2030181	**2.63E-05**	0.01125215	-11.7%	3a
HVR1	0.22916202	0.08168042	2.80559316	**0.00502241**	0.01602382	25.8%	3a
E2	0.06924998	0.02166032	3.19708955	**0.00138822**	0.0136558	7.2%	3a

P7	0.08908964	0.04628874	1.92465039	**0.05427311**	0.01392944	9.3%	3a
NS2	0.0443179	0.02632395	1.68355821	0.09226708	0.01331955	4.5%	3a
NS5B	-0.0953281	0.02336351	-4.0802136	**4.50E-05**	0.01158356	-9.1%	3a
t:Nonsyn	-0.1553399	0.0398978	-3.8934443	**9.88E-05**	0.01090885	-14.4%	3a
c:Nonsyn	-0.1388131	0.04132345	-3.3591858	**0.00078172**	0.01109064	-13.0%	3a
g:Nonsyn	0.04033366	0.04326127	0.93232714	0.35116749	0.01326658	4.1%	3a

Table S4. Proportions and frequencies of observed known resistance associated variants (RAVs) assessed in our study. (A) Results summarized by subtypes, and (B) by gene for each subtype.

A

Subtype	% samples with RAVs	Ave freq.(non-fixed RAVs only)	% of RAVs with fixed RAVs	Ave % of samples with fixed RAVs per RAV
1a	65.8%	0.0031	25.3%	5.66%
1b	72.8%	0.0032	12.3%	17.1%
3a	58.5%	0.0017	64.0%	4.28%

B

	Gene	1a	1b	3a
% samples with RAVs	NS3	70.2%	74.2%	58.9%
	NS5A	63.8%	73.9%	58.2%
	NS5B	54.0%	63.0%	57.5%
Average freq.	NS3	0.0032	0.0028	0.0015
	NS5A	0.0036	0.0044	0.0020
	NS5B	0.0017	0.0022	0.0017
Ave. % samples with fixed RAVs	NS3	8.2%	14.7%	4.2%
	NS5A	2.5%	17.9%	4.7%
	NS5B	0	0	2.6%

Fig. S1. Relationship between viral load and nucleotide diversity (π) or average read depth of all HCV samples used in our analyses. (A) Viral load vs. nucleotide diversity (Spearman's correlation test, *P*-value = 0.9351, rho = 0.00644). (B) Viral load vs. average read depth (Spearman's correlation test, *P*-value = 0.168, rho = -0.11).

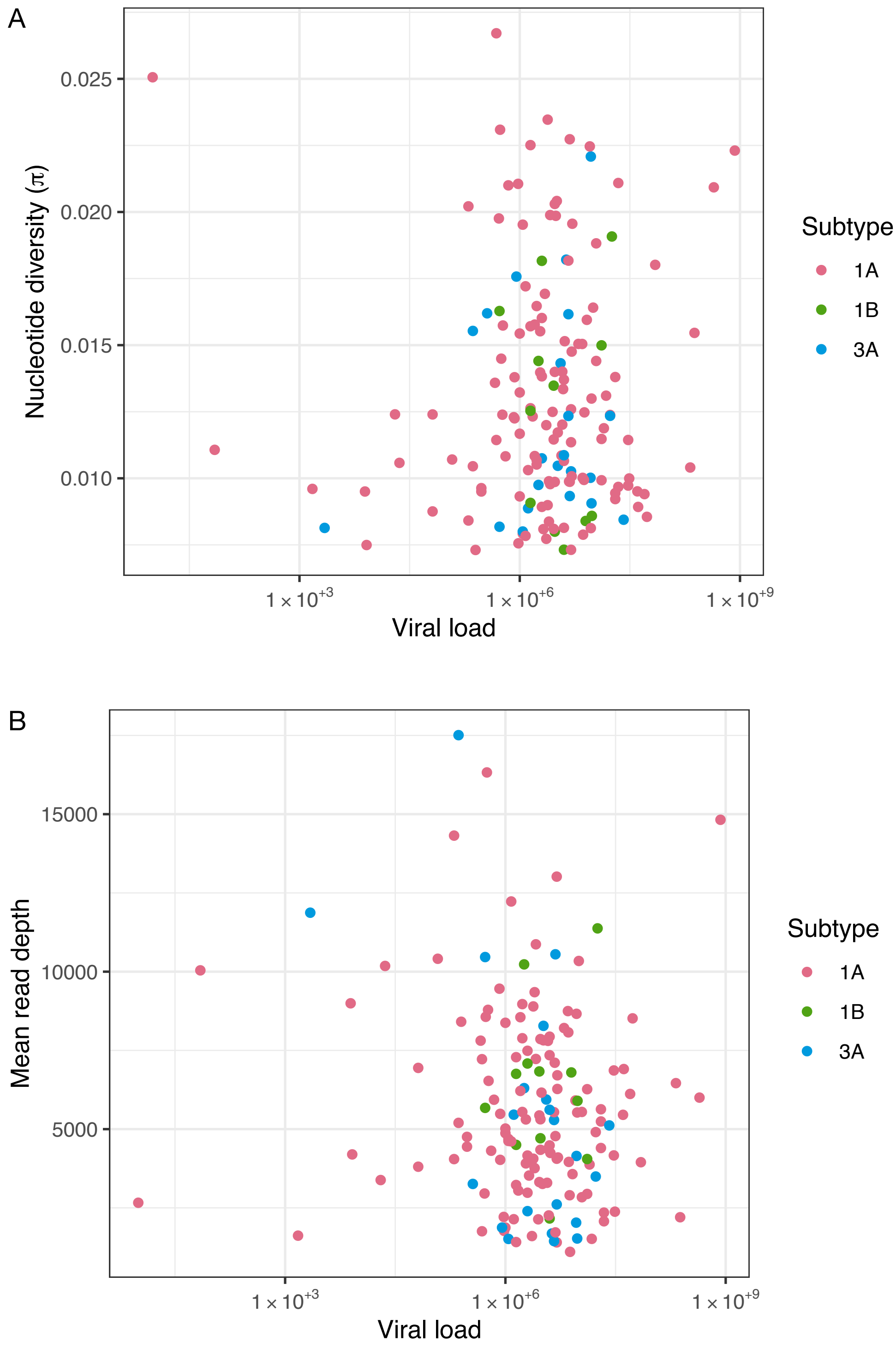

Fig. S2. Nucleotide diversity (π) and fixation indices (F_{ST}) between HCV subtypes. Upper diagonal represents nucleotide diversity of two subtypes combined, values on diagonal are nucleotide diversity within each subtype, and lower diagonal represents F_{ST} values between the two subtypes.

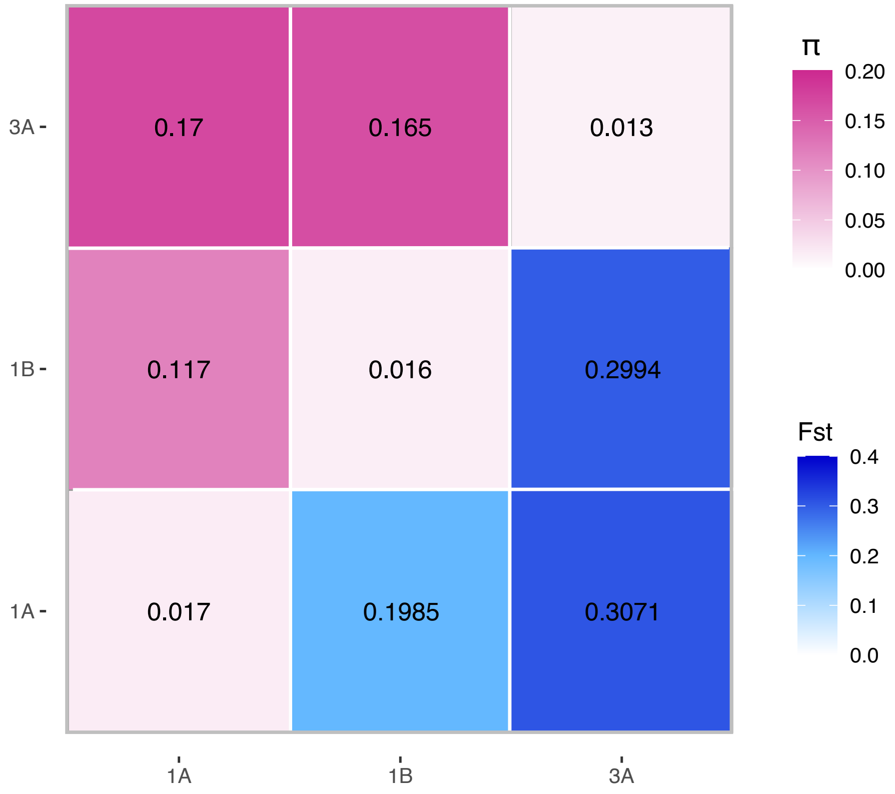

Fig. S3. Between-host nucleotide diversity (π) of each subtype. Nucleotide diversity were calculated separately for synonymous sites (syn) and nonsynonymous sites (nonsyn).

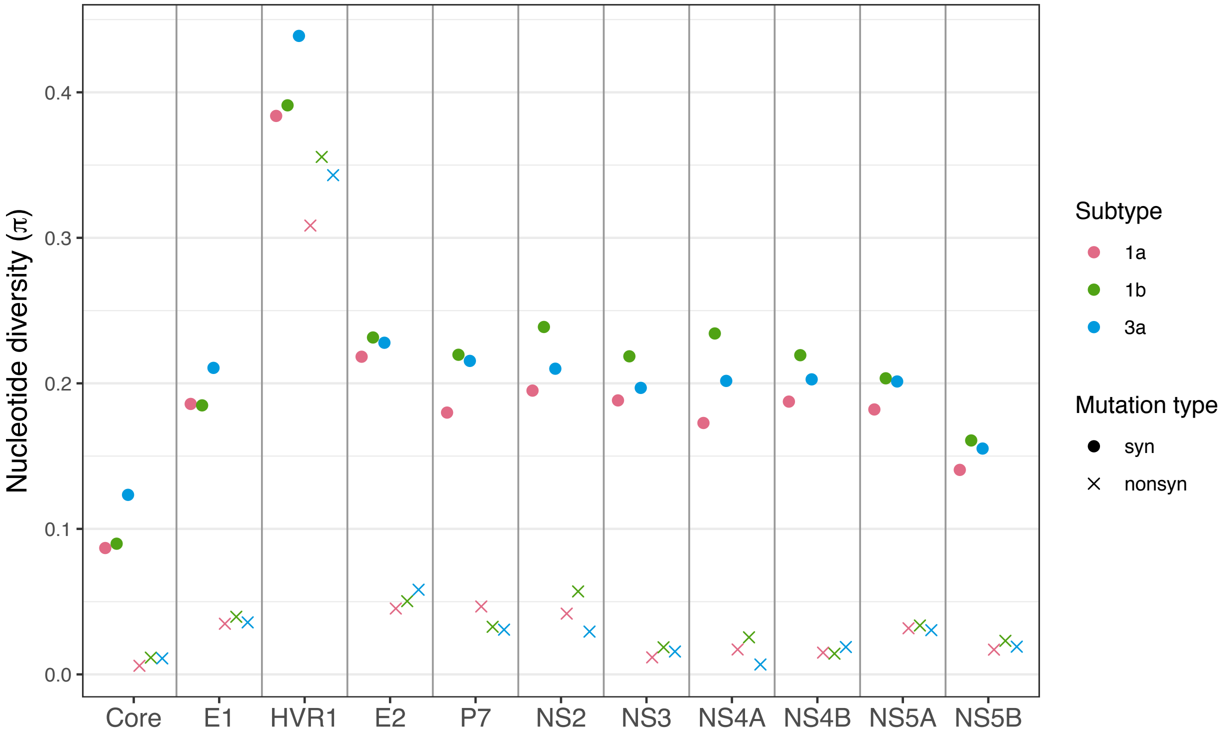

Supplement: Supplementary file 1 [file viruses-13-00511-s001.zip › SupplementaryTablesFigures.pdf]
